# Anti-inflammatory effects of Frondanol, a nutraceutical extract from *Cucumaria frondosa*, via modulation of NF-κB and MAPK pathways in LPS-induced RAW 264.7 cells

**DOI:** 10.3389/fphar.2025.1683630

**Published:** 2025-10-30

**Authors:** Hardik Ghelani, Kerat Hanspal, Lana Talo, Sami Talo, Hala Altaher, Hadil Sarsour, Marah Tabbal, Sally Badawi, Peter Collin, Thomas E. Adrian, Reem K. Jan

**Affiliations:** ^1^ College of Medicine, Mohammed Bin Rashid University of Medicine and Health Sciences, Dubai, United Arab Emirates; ^2^ Coastside Bio Resources, Deer Isle, ME, United States

**Keywords:** Frondanol, sea cucumber, inflammation, NF-κB, MAPK, RAW 264.7 macrophage

## Abstract

**Introduction:**

Frondanol, a non-polar lipid extract derived from the edible sea cucumber Cucumaria frondosa, has shown promising anti-inflammatory properties.

**Methods:**

This study investigated its molecular mechanisms in modulating inflammation using lipopolysaccharide (LPS)-stimulated RAW 264.7 murine macrophages.

**Results:**

Frondanol was found to be non-cytotoxic at the tested dilutions (1:80,000 to 1:10,000). Co-treatment with LPS and Frondanol significantly reduced the production of inflammatory mediators. Nitric oxide (NO) levels were decreased by up to 30% (p < 0.05), while iNOS protein and mRNA expression were reduced by approximately 45% (p < 0.05) and 80% (p < 0.0001), respectively, at a 1:10 K dilution. Prostaglandin E_2_ (PGE_2_) levels were suppressed by nearly 40% (p < 0.0001), accompanied by a 60% reduction in COX-2 protein (p < 0.01) and a 70% decrease in COX-2 mRNA expression (p < 0.05). The pro-inflammatory cytokines including tumor necrosis factor (TNF)-α, interleukin (IL)-1β, and interleukin (IL)-6 were also significantly attenuated by Frondanol treatment. Mechanistically, Frondanol inhibited LPS-induced NF-κB activation by reducing IκBα phosphorylation and preventing nuclear translocation of NF-κB p65. Furthermore, Frondanol significantly downregulated the phosphorylation of mitogen-activated protein kinases (MAPKs), including ERK1/2, JNK, and p38.

**Conclusion:**

These results suggest that Frondanol exerts its anti-inflammatory effects through suppression of both NF-κB and MAPK signalling pathways, leading to reduced production of inflammatory mediators and cytokines. Given its efficacy and lack of cytotoxicity, Frondanol may hold strong potential as a nutraceutical agent for the prevention and management of chronic inflammatory diseases.

## 1 Introduction

Inflammation is a physiological response of the immune system to various agents, including infections and toxins, involving the overproduction of inflammatory cytokines (Che et al., 2018). Although this response is crucial in regulating tissue repair and inflammation, the excess generation of these mediators contributes to the development of inflammatory diseases (Soliman and Barreda, 2022; Kany et al., 2019). Therefore, it is essential to control inflammation to prevent and treat such conditions. Macrophages play a significant role in the host defence mechanism and are commonly activated in inflamed tissues following exposure to interferon-γ (IFN-γ), tumor necrosis factor (TNF)-α, or microbial lipopolysaccharides (LPS) (Klein et al., 2019; Chawla et al., 2011). Once activated, macrophages produce pro-inflammatory cytokines such as TNF-α, interleukin (IL)-1β, and interleukin (IL)-6, as well as inflammatory mediators such as nitric oxide (NO), prostaglandin E2 (PGE2), and leukotriene (LT)-B4 (Parisi et al., 2018). Their synthesis and release at high levels are implicated in the development of chronic diseases such as atherosclerosis (Kong et al., 2022), rheumatoid arthritis (Bedeković et al., 2023), inflammatory bowel disease (IBD) (Neurath, 2014), and cancer (Singh et al., 2019).

A key factor in macrophage activation is LPS, which are endotoxins derived from Gram-negative bacteria. LPS are large amphipathic glycoconjugates consisting of a hydrophobic lipid domain attached to a core oligosaccharide and a distal polysaccharide. LPS stimulate macrophages through Toll-like receptor 4 (TLR4), triggering the activation of several inflammatory signalling pathways, including nuclear factor-κB (NF-κB) and mitogen-activated protein kinases (MAPK). This cascade results in the excessive production of inflammatory mediators and cytokines, culminating in abnormal inflammatory responses that contribute to the pathogenesis of various inflammatory diseases (Kuzmich et al., 2017). Under basal conditions, NF-κB is kept inactive in the cytoplasm by its inhibitor, nuclear factor of kappa light polypeptide gene enhancer in B-cells inhibitor alpha (IκB-α). Upon exposure to LPS or other inflammatory stimuli, IκB-α is degraded, allowing NF-κB to translocate to the nucleus and initiate the transcription of genes involved in the inflammatory response (Liu et al., 2017). In addition to NF-κB, kinases such as MAPK—including extracellular signal-regulated kinase (ERK), c-Jun N-terminal kinase (JNK), and p38 MAPK—along with the PI3K/Akt pathway, contribute to the regulation of cytokine expression and further amplify NF-κB activation in macrophages (Dorrington and Fraser, 2019; Moens et al., 2013).

Given the critical role of these inflammatory pathways in diseases such as atherosclerosis, rheumatoid arthritis, IBD, and cancer, targeting these mechanisms has become a promising strategy for developing new or adjunct anti-inflammatory therapies (Zhao et al., 2021). Marine-sourced natural products, particularly polysaccharides derived from marine microorganisms, have shown significant potential in modulating inflammatory pathways (Cheung et al., 2016; Notarte et al., 2018; Notarte et al., 2017). These bioactive compounds can inhibit the activation of NF-κB and other inflammatory signalling pathways, making them attractive candidates for the prevention and treatment of chronic inflammatory diseases (Cheung et al., 2016; Yasir et al., 2024).

Sea cucumbers and their extracts are known for their high nutritional value and various potential health benefits, including anti-inflammatory effects. For centuries, they have been used as customary foods and in folk medicine in countries including Japan, Indonesia, Korea, and China (Ghelani et al., 2022). Frondanol, a nutraceutical non-polar extract of the edible sea cucumber *Cucumaria frondosa*, is a US-patented agent reported to possess potent anti-inflammatory activity, observed in animal and human studies without signs of toxicity (Ghelani et al., 2023; Subramanya et al., 2018; Collin, 2002). In a murine model of colitis, Frondanol administration resulted in significant reductions in colonic inflammation and inflammatory cytokines, suggesting immunomodulatory effects within the gut mucosa (Subramanya et al., 2018). Moreover, *in vitro* studies have shown that Frondanol inhibits the 5-lipoxygenase (5-LOX) and 12-lipoxygenase (12-LOX) pathways, reducing the production of lipid mediators such as 12-hydroxyeicosatetraenoic acid (12-HETE), 5-hydroxyeicosatetraenoic acid (5-HETE), and leukotriene B4 (LTB4) in human neutrophils (Collin, 2002).

Building on these findings, a randomized, double-blind, placebo-controlled clinical trial was designed to evaluate Frondanol’s efficacy in adults with IBD (Ghelani et al., 2023). This published protocol outlines the study design, inclusion criteria, and endpoints, and unpublished preliminary findings support potential anti-inflammatory effects in patients with IBD, suggesting clinical relevance of the observations seen in colitis model of IBD. While the existing literature supports Frondanol’s anti-inflammatory potential, the mechanism(s) by which it exerts these effects remains largely unknown, particularly in macrophage-driven inflammation. Therefore, this study aimed to investigate the molecular mechanisms underlying the anti-inflammatory activity of Frondanol on a cellular model of LPS-induced RAW 264.7 macrophages. We hypothesized that Frondanol attenuates LPS-induced inflammatory responses by modulating key signaling pathways, specifically NF-κB and MAPK. To test this, we evaluated its effects on the production of inflammatory mediators, including nitric oxide and prostaglandin E_2_, the expression of inducible enzymes such as iNOS and COX-2, and the secretion of pro-inflammatory cytokines (TNF-α, IL-1β, and IL-6), as well as the phosphorylation status of key proteins involved in NF-κB and MAPK pathways.

## 2 Materials and methods

### 2.1 Preparation of Frondanol

Frondanol is a trademarked nutraceutical lipid extract (developed and marketed by Coastside BioResources, Stonington, ME, United States) derived from the gut material of edible Atlantic Sea cucumber (*Cucumaria frondosa).* The extraction process involves using hexane as an organic solvent to isolate lipids from the intestinal tissues of the sea cucumber. This extract is used in various dietary supplements and health products due to its purported health benefits. Frondanol, in the form of oil, is encapsulated within soft-gel capsules. For the current investigation, the oil contained in the capsules was initially dissolved in dimethyl sulfoxide (DMSO) to prepare a working solution. This DMSO-dissolved oil was then further diluted in culture media to produce a series of dilutions: 1:10,000, 1:20,000, 1:40,000, and 1:80,000, corresponding to final concentrations of 0.01%, 0.005%, 0.0025%, and 0.00125% (v/v), respectively. To achieve maximum solubility, each dilution step was carried out using warm (37 °C) conditions, followed by vigorous mixing and brief sonication to ensure the Frondanol was completely solubilized.

### 2.2 Gas chromatography-flame ionization total fatty acid analysis of Frondanol

The fatty acid profile of Frondanol was determined by Eurofins Scientifics (United States) using gas chromatography with flame ionization detection (GC-FID). Fatty acids were first liberated from triglycerides and complex lipids through saponification and subsequently converted into fatty acid methyl esters (FAMEs) via transesterification. An internal standard (e.g., C13:0 or C19:0) was added before extraction to ensure accurate quantification. FAMEs were separated using a wax-type capillary column, and peaks were identified and quantified based on retention times. Results were reported as weight percent of individual fatty acids relative to the total sample. This method allowed the quantification of saturated, monounsaturated, and polyunsaturated fatty acids, such as EPA and DHA.

### 2.3 Cell culture and viability assay

Murine macrophage RAW 264.7 cells, purchased from Addex Bio (San Diego, CA United States), were cultured at 37 °C in DMEM supplemented with 10% FBS, penicillin (100 units/mL), and streptomycin (100 μg/mL) under a humidified atmosphere of 5% CO2. The cell viability was determined using 3-(4,5-dimethylthiazol-2-yl)-2,5-diphenyl tetrazolium bromide (MTT) assay. Briefly, 1 × 10^5^ cells/well seeded in a 96-well plate in 200 μL cell culture medium. After 24 h, cells were treated with different dilutions (1:80,000, 1:40,000, 1:20,000, and 1:10,000 (v/v)) of Frondanol and various concentrations (0.0625%, 0.12%, 0.25% and 0.5%) of DMSO (representing DMSO concentration in each Frondanol dilution) for 24 h, and cells without treatment served as the control. After 24 h of treatment, cells were incubated with 5 mg/mL MTT solution for 4 h at 37 °C and 5% CO2. Then, the medium was removed, and 150 μL DMSO was added to dissolve the precipitate. Finally, the absorbance (OD) was measured at 570 nm in a microplate reader. The relative cell viability was calculated using the following equation:

$$\text{Cell viability }\left(\%\right)=\frac{\text{OD}\;\text{of}\;\text{experimental}\;\text{group}}{\text{OD}\;\text{of}\;\text{control group}\;}\;X\;100$$

Frondanol, being a non-polar lipid extract, was dissolved in DMSO to ensure proper solubilization. The highest concentration of Frondanol tested (1:10 K dilution) corresponded to a final DMSO concentration of 0.5%, which was used as the vehicle control in all experiments. Preliminary cell viability assays confirmed that 0.5% DMSO was non-cytotoxic to RAW 264.7 cells. Therefore, 0.5% DMSO was included as a negative control to distinguish the specific effects of Frondanol from any potential influence of the solvent.

### 2.4 Determination of nitric oxide (NO) production

RAW 264.7 cells were seeded on 96-well plates (2 × 10^5^ cells/well) and cultured for 24 h. After that, cells were co-treated with LPS (1 μg/mL) alone and with different dilutions of Frondanol for 24 h. DMSO (final concentration of 0.5% (v/v)) served as a negative control, and cells without treatment served as the control, while cells treated with LPS alone served as LPS-model control. After 24 h of treatment, 100 μL of cell culture supernatant was collected from each well and mixed with an equal volume of Griess reagent. The mixture was incubated for 15 min, and absorbance was then measured at 540 nm. The final concentration of NO in each well was determined using a nitrite standard curve.

### 2.5 Determination of PGE2, IL-6, IL-1β and TNF-α in cell supernatant

The cytokine production induced by LPS stimulation in RAW 264.7 macrophages was determined using respective mouse ELISA kits. Briefly, cells were seeded in 96-well plates (2 × 10^5^ cells/well) and cultured for 24 h. After that, cells were co-treated with LPS (1 μg/mL) alone and with various dilutions of Frondanol for 24 h. DMSO (final concentration of 0.5% (v/v)) served as a negative control, and cells without treatment served as the control, while cells treated with LPS alone served as LPS-model control. After 24 h of treatment, 100 μL of cell culture supernatant was collected from each well, and cytokines were measured following the manufacturer’s protocols for each ELISA kit.

### 2.6 Preparation of cytosolic and nuclear extracts

RAW 264.7 cells (2 × 10^6^ cells/10 cm dish) were seeded and pretreated with two different dilutions of Frondanol (1:20 K and 1:10 K) for 4 h. They were then stimulated with LPS (1 μg/mL) for 30 min. Thereafter, cells were washed twice with cold PBS and harvested with 1 mL of PBS. Cell pellets were suspended in 150 μL of hypotonic buffer (10 mM HEPES/KOH, 10 mM KCl, 2 mM MgCl2, 0.1 mM EDTA, 1 mM DTT, and 0.5 mM PMSF, pH 7.9) and incubated on ice for 15 min. Cells were homogenized by passing through a 1 mL syringe using a 27-gauge needle (at least ten times). Cell homogenates were spun at 14,000 x g for 15 min at 4 °C. The supernatant was collected and used as cytosolic fraction while the pellet was gently resuspended in 50 μL of complete lysis buffer (50 mM HEPES/KOH, 50 mM KCl, 1 mM DTT, 300 mM NaCl, 0.1 mM EDTA, 10% glycerol, and 0.5 mM PMSF, pH 7.9) and centrifuged at 13000 *g* for 20 min at 4 °C. The supernatant was used as the nuclear extract.

### 2.7 Preparation of complete cell lysate

RAW 264.7 cells co-treated with LPS (1 μg/mL) alone and with different dilutions of Frondanol for 24 h. DMSO (final concentration of 0.5% (v/v)) served as a negative control, and cells without treatment served as the control, while cells treated with LPS alone served as LPS-model control. After 24 h treatments, cells were harvested and suspended in RIPA lysis buffer containing a phosphatase and protease inhibitor cocktail and centrifuged at 14,000 rpm, at 4 °C, for 25 min to separate cell proteins. The supernatant was used to determine the protein expression of iNOS and COX-2 using the Western blot method.

### 2.8 Western immunoblot analysis

The extracted proteins (cytosolic, nuclear, or whole cell lysate) were quantified through a Pierce™ BCA Protein assay kit (Thermo Fisher, MA, United States), and 20–50 µg was electrophoresed on an acrylamide gel (4%–15% Mini-PROTEAN^®^ TGX™ Precast; Bio-Rad, California, United States). The separated proteins were transferred to a nitrocellulose membrane. Then, the membrane and various primary antibodies: anti-COX-2, anti-iNOS, anti-IκB-α, anti-phopspho-IκB-α, anti-NF-κB, anti-ERK1/2, anti-phospho-ERK1/2, anti-JNK, anti-phospho-JNK, anti-P38, anti-phospho-P38, anti-GAPDH and anti-Histon H3, were left to react overnight at 4 °C on the shaker. After that, the membrane was left to react with the specific secondary antibody for 1 h at room temperature; then, it was washed sufficiently, and the protein expression level was obtained using a chemiluminescent substrate (Thermo Fisher, MA, United States). Western blot results were quantified using ImageJ (National Institutes of Health, Bethesda, MD, United States).

### 2.9 Real-time reverse transcription-polymerase chain reaction

RAW 246.7 cells were seeded in six-well plates and co-treated with LPS (1 μg/mL) alone and with different dilutions of Frondanol for 24 h. Following treatment, cells were harvested by trypsinization, and total RNA was isolated using the Relia Prep™ RNA Cell Miniprep System (Promega, United States) and conducted according to the manufacturer’s protocol. The RNA underwent evaluation for its quality and quantity using a Nanodrop spectrophotometer, after which purified RNA samples were preserved at −80 °C until required. The cDNA synthesis was conducted using the High-Capacity cDNA Archive kit (Applied Biosystems, MA, United States). A mixture comprising a sample of cDNA (2 µL), forward and reverse primers (each 0.5 µL; sequences provided in Table 1), Power SYBER Green PCR master mix (5 µL), and nuclease-free water (4 µL) was prepared, resulting in a total reaction volume of 12 µL. These reactions were conducted using a Step One Plus PCR apparatus (Thermo Fisher Scientific). Reaction conditions involved 40 cycles at 95 °C for 35 s, 55–59 °C for 30 s, 95 °C for 15 s, and 60 °C for 60 s, followed by a final extension at 95 °C for 15 s. The cDNA samples for each gene underwent two parallel amplifications, and the expression level of the target gene was determined as the mean of the Ct values. The housekeeping gene β-actin served as an internal reference.

**TABLE 1:** The primers used for real-time reverse transcription-polymerase chain reaction analysis.

Gene	Sequence
*iNOS*	F – TGG​AGC​GAG​TTG​TGG​ATT​GT
	R - CTC​TGC​CTA​TCC​GTC​TCG​TC
*COX-2*	F - ACC​TGG​TGA​ACT​ACG​ACT​GC
	R- TGG​TCG​GTT​TGA​TGT​TAC​TG
*IL-1*β	F - GGG​CCT​CAA​AGG​AAA​GAA​TC
	R - TAC​CAG​TTG​GGG​AAC​TCT​GC
*IL-6*	F - GTT​GCC​TTC​TTG​GGA​CTG​AT
	R – CAT​TTC​CAC​GAT​TTC​CCA​GA
*TNF-*α	F – GCG​ACG​TGG​AAC​TGG​CAG​AA
	R - CAG​TAG​ACA​GAA​GAG​CGT​GGT​G
*β-actin*	F – TGCTGTCCCTGTATGCCTCTG R - GCTGTAGCCACGCTCGGTCA

### 2.10 Statistical analysis

Statistical analyses were conducted using GraphPad Prism (version 10.2.1.) soft-ware (San Diego, CA, United States). Differences among treatment groups and untreated groups were assessed using a one-way analysis of variance (ANOVA) followed by Dunnett’s *post hoc* multiple comparison test. The significance level of p < 0.05 was considered statistically significant.

## 3 Results

### 3.1 Fatty acid profiling of Frondanol

Fatty acid profiling of Frondanol revealed the highest concentrations of fatty acids to be 12-methyltetradecanoic acid (12-MTA, 19.6%), followed by eicosapentaenoic (EPA, 17.9%), palmitoleic acid (12.7%), oleic acid (4.49%), stearic acid (2.84%), myristoleic acid (2.71%), myristic (2.54%), linoleic acid (2.54%) and docosahexaenoic acid (DHA, 0.6%) (Table 2). The overall composition included 9.52% saturated fatty acids, 21.68% monounsaturated, and 23.73% polyunsaturated fatty acids (Table 3). This bioactive lipid profile revealed that it is rich in the branched-chain fatty acid (12-MTA) as well as long-chain polyunsaturated fatty acids such as EPA and DHA, all of which are known for their immunomodulatory and anti-inflammatory properties.

**TABLE 2:** Fatty acid profile of Frondanol.

Fatty acid	% Weight
12-Methyltetradecanoic acid (12-MTA)	19.6
C08:0 Octanoic (Caprylic)	<0.1
C10:0 Decanoic (Capric)	<0.1
C11:0 Undecanoic (Hendecanoic)	<0.1
C12:0 Dodecanoic (Lauric)	<0.1
C14:0 Tetradecanoic (Myristic)	2.54
C14:1 Tetradecenoic (Myristoleic)	2.71
C15:0 Pentadecanoic	1.07
C15:1 Pentadecenoic	<0.1
C16:0 Hexadecanoic (Palmitic)	1.97
C16:1 Hexadecenoic (Palmitoleic)	12.7
C16:2 Hexadecadienoic	<0.1
C16:3 Hexadecatrienoic	<0.1
C16:4 Hexadecatetraenoic	<0.1
C17:0 Heptadecanoic (Margaric)	0.56
C17:1 Heptadecenoic Margaroleic	<0.1
C18:0 Octadecanoic (Stearic)	2.84
C18:1 Octadecenoic (Oleic)	4.49
C18:2 Octadecadienoic (Linoleic)	2.54
C18:3 Octadecatrienoic (Linolenic)	0.28
C18:4 Octadecatetraenoic	<0.1
C19:0 Nonadecanoic	<0.1
C20:0 Eicosanoic (Arachidic)	0.33
C20:1 Eicosenoic (Gadoleic)	0.39
C20:2 Eicosadienoic	<0.1
C20:3 Eicosatrienoic	0.55
C20:4 Eicosatetraenoic (Arachidonic)	0.60
C20:5 Eicosapentaenoic (EPA)	17.9
C21:0 Heneicosanoic	<0.1
C21:5 Heneicosapentaenoic	<0.1
C22:0 Docosanoic (Behenic)	0.21
C22:1 Docosenoic (Erucic)	0.30
C22:2 Docosadienoic	0.80
C22:3 Docosatrienoic	<0.1
C22:4 Docosatetraenoic	<0.1
C22:5 Docosapentaenoic	0.46
C22:6 Docosahexaenoic (DHA)	0.60
C24:0 Tetracosanoic (Lignoceric)	<0.1
C24:1 Tetracosenoic (Nervonic)	0.99
Unidentified Fatty acids	25.6

**TABLE 3:** Fatty acids composition in Frondanol.

Fatty acid composition	% Weight
Saturated	9.52
Monosaturated	21.68
Polyunsaturated	23.73

### 3.2 Cell viability

**FIGURE 1:**
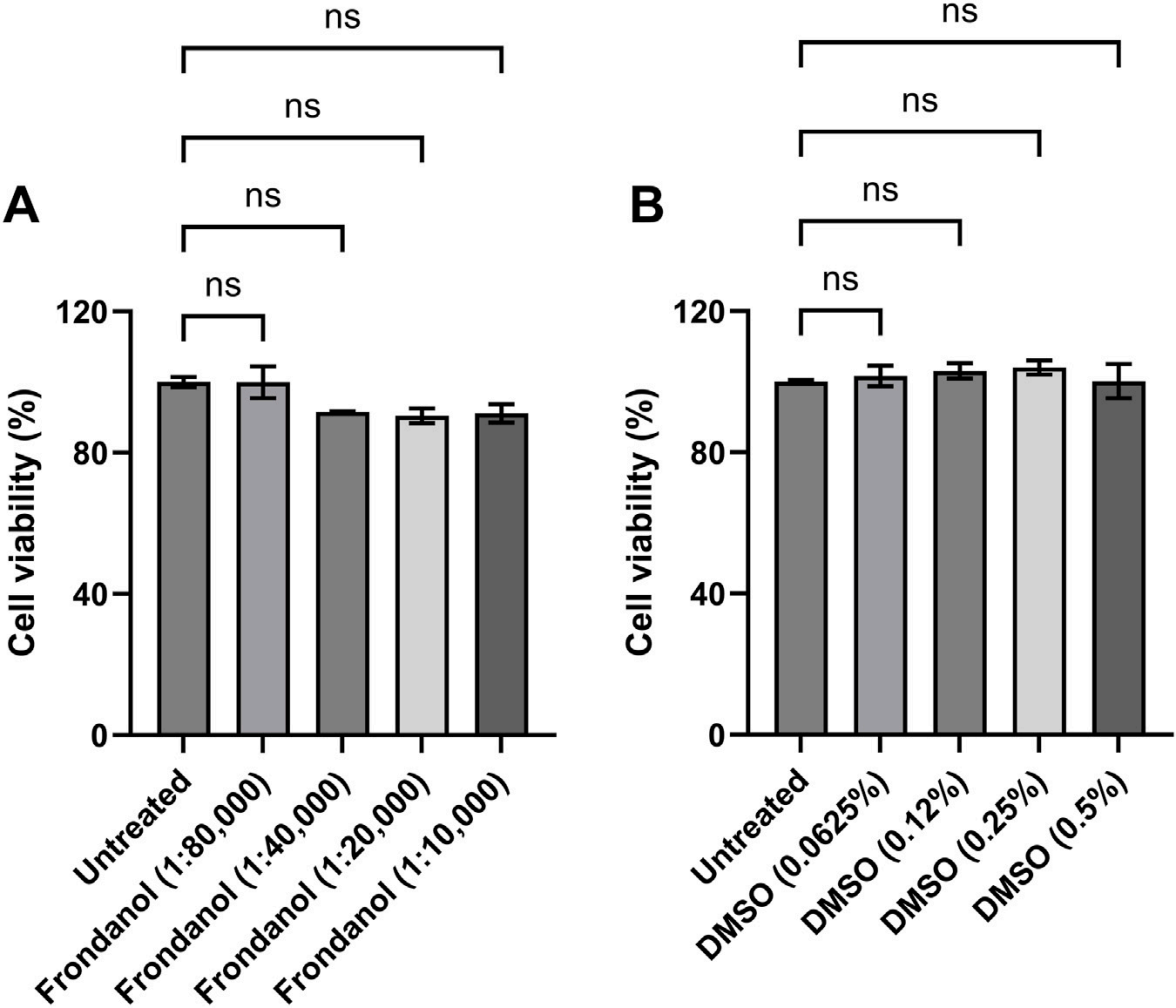
Effects of Frondanol and DMSO on the viability of RAW 264.7 macrophage. The percentage cell viability of RAW 264.7 macrophages treated with **(A)** Frondanol **(B)** DMSO treatment. Each bar represents the mean ± SEM of three independent experiments. ns = non-significant when compared to untreated.

RAW 264.7 macrophages were treated with different concentrations of Frondanol (1:80 K, 1:40 K, 1:20 K, and 1:10 K) for 24 h. As shown in Figure 1, none of the tested Frondanol concentrations demonstrated any toxicity to the cells, indicating that these concentrations are safe for use. Therefore, Frondanol concentrations between 1:80 K and 1:10 K were selected for subsequent experiments. Additionally, we examined the effect of DMSO on cell viability. The DMSO concentrations corresponding to the Frondanol dilutions (0.0625%, 0.125%, 0.25%, and 0.5%) did not affect cell viability as well, as illustrated in Figure 1.

### 3.3 Frondanol attenuates LPS-induced NO production, iNOS protein, and gene expression in RAW 264.7 macrophages

**FIGURE 2:**
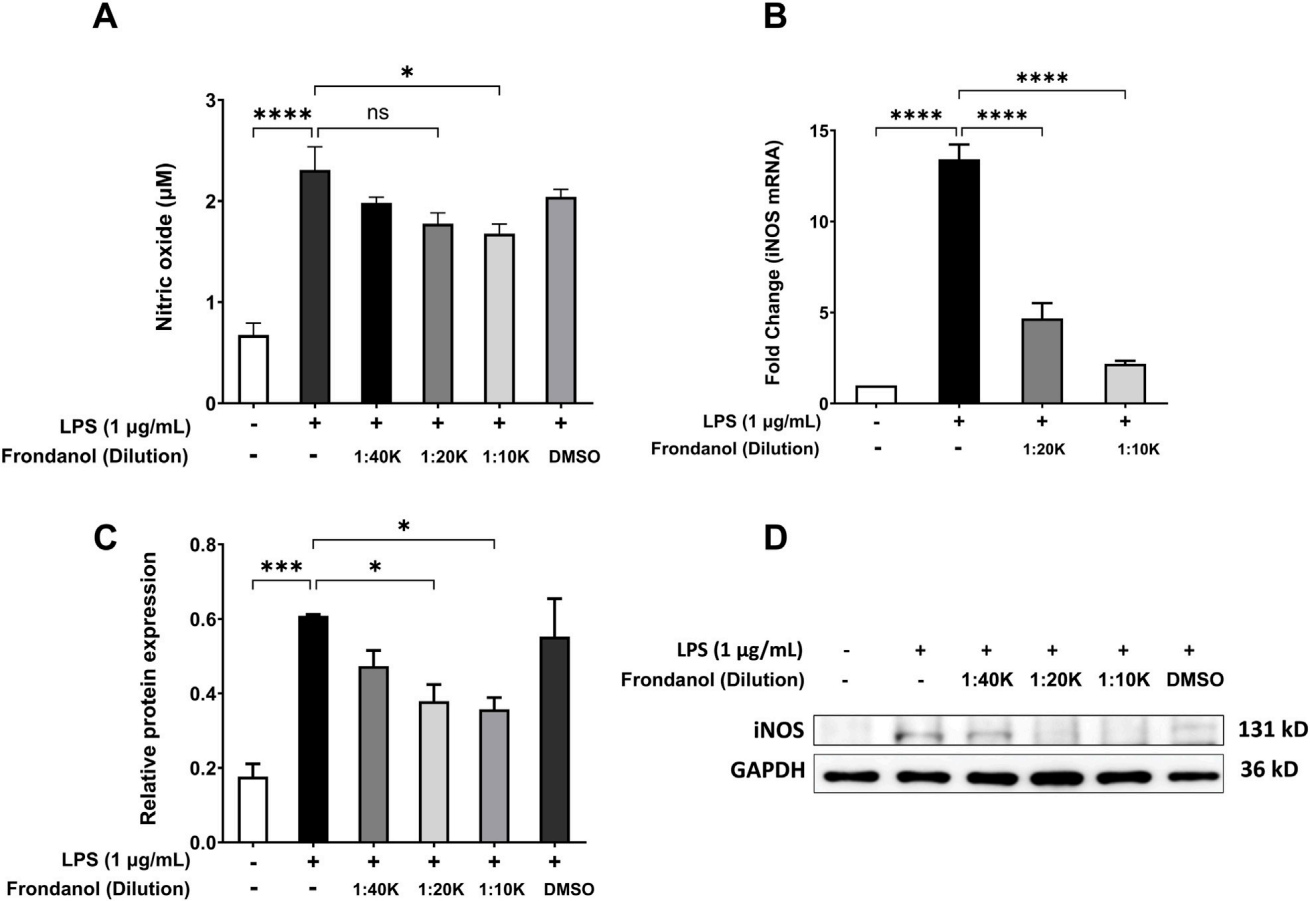
Effects of Frondanol on **(A)** nitric oxide production, **(B)** inducible nitric oxide synthase mRNA levels (qPCR), and **(C,D)** protein expression (Western blot) under different treatment conditions in RAW 264.7 macrophages. Data are mean ± SEM of three independent experiments. *p < 0.05, **p < 0.01, ***p < 0.001, and ****p < 0.0001.

As shown in Figure 2A, LPS stimulation significantly increased NO production compared to the control group (p < 0.001). Treatment with Frondanol at 1:20 K and 1:10 K dilutions significantly reduced NO production in LPS-stimulated RAW 264.7 macrophages (p < 0.05). Additionally, we measured the gene and protein expression of iNOS, which is responsible for NO synthesis in RAW 264.7 macrophages. Western blot analysis (Figures 2C,D) showed that LPS markedly upregulated iNOS protein expression compared to the control. Frondanol treatment led to a significant reduction in iNOS protein levels at 1:20 K and 1:10 K dilutions (p < 0.05), indicating a dose-dependent inhibitory effect. Since the 1:40 K dilution did not show a marked reduction in iNOS expression at the protein level, it was excluded from subsequent qPCR analysis. As shown in Figure 2B, the mRNA levels of iNOS, measured by qPCR, were significantly upregulated by LPS treatment (p < 0.001). Frondanol treatment at 1:20 K and 1:10 K dilutions led to a significant reduction in iNOS mRNA expression, with 1:20 K showing a reduction (p < 0.01) and 1:10 K showing an even more pronounced reduction (p < 0.001) compared to the LPS model group.

### 3.4 Frondanol attenuates LPS-induced PGE2 production, COX-2 protein, and gene expression in RAW 264.7 macrophages

**FIGURE 3:**
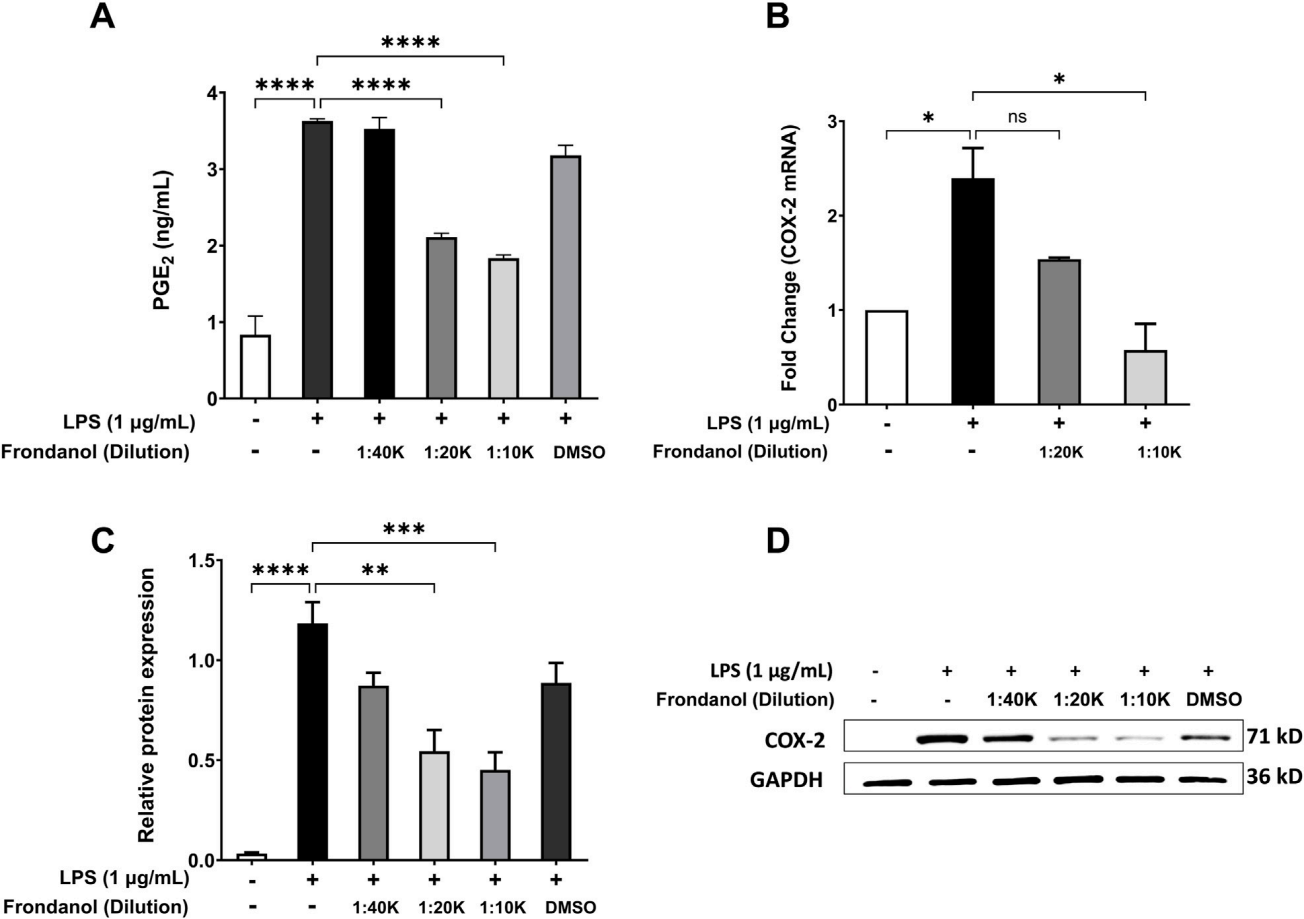
Effects of Frondanol on **(A)** prostaglandin E2 production, **(B)** cyclooxygenase-2 mRNA levels (qPCR), and **(C,D)** protein expression (Western blot) under different treatment conditions in RAW 264.7 macrophages. Data are mean ± SEM of three independent experiments. *p < 0.05, **p < 0.01 and ***p < 0.001, ****p < 0.0001.

LPS treatment (1 μg/mL) significantly increased the production of PGE2 compared to the untreated control (p < 0.0001). Treatment with Frondanol at dilutions of 1:40 K, 1:20 K, and 1:10 K caused significant concentration dependent reduction in PGE2 levels compared to the LPS model group (p < 0.0001). DMSO treatment did not significantly affect PGE2 levels compared to the LPS model group (Figure 3A). Additionally, we measured the mRNA and protein expression of COX-2, which is responsible for PGE2 production in RAW 264.7 macrophages. The Western blot analysis revealed that LPS treatment significantly increased COX-2 protein expression compared to the untreated control (p < 0.0001). Frondanol treatment at 1:20 K, and 1:10 K dilutions significantly reduced COX-2 expression levels compared to the LPS model group (p < 0.01 and p < 0.001, respectively). The most substantial decrease in COX-2 expression was observed at the 1:10 K dilution, confirming the dose-dependent effect of Frondanol. The DMSO-treated group did not significantly differ from the LPS model group. Since the 1:40 K dilution did not show a marked reduction in COX-2 expression at the protein level, it was excluded from subsequent qPCR analysis. The fold change analysis demonstrated that LPS exposure significantly altered the mRNA expression of COX-2 compared to the untreated control group. Similarly, treatment with Frondanol at 1:10 K dilution led to significant changes in the mRNA expression of COX-2 compared to the LPS model group (p < 0.05) (Figure 3B).

### 3.5 Frondanol attenuates LPS-induced pro-inflammatory cytokine secretion and gene expression in RAW 264.7 macrophages

**FIGURE 4:**
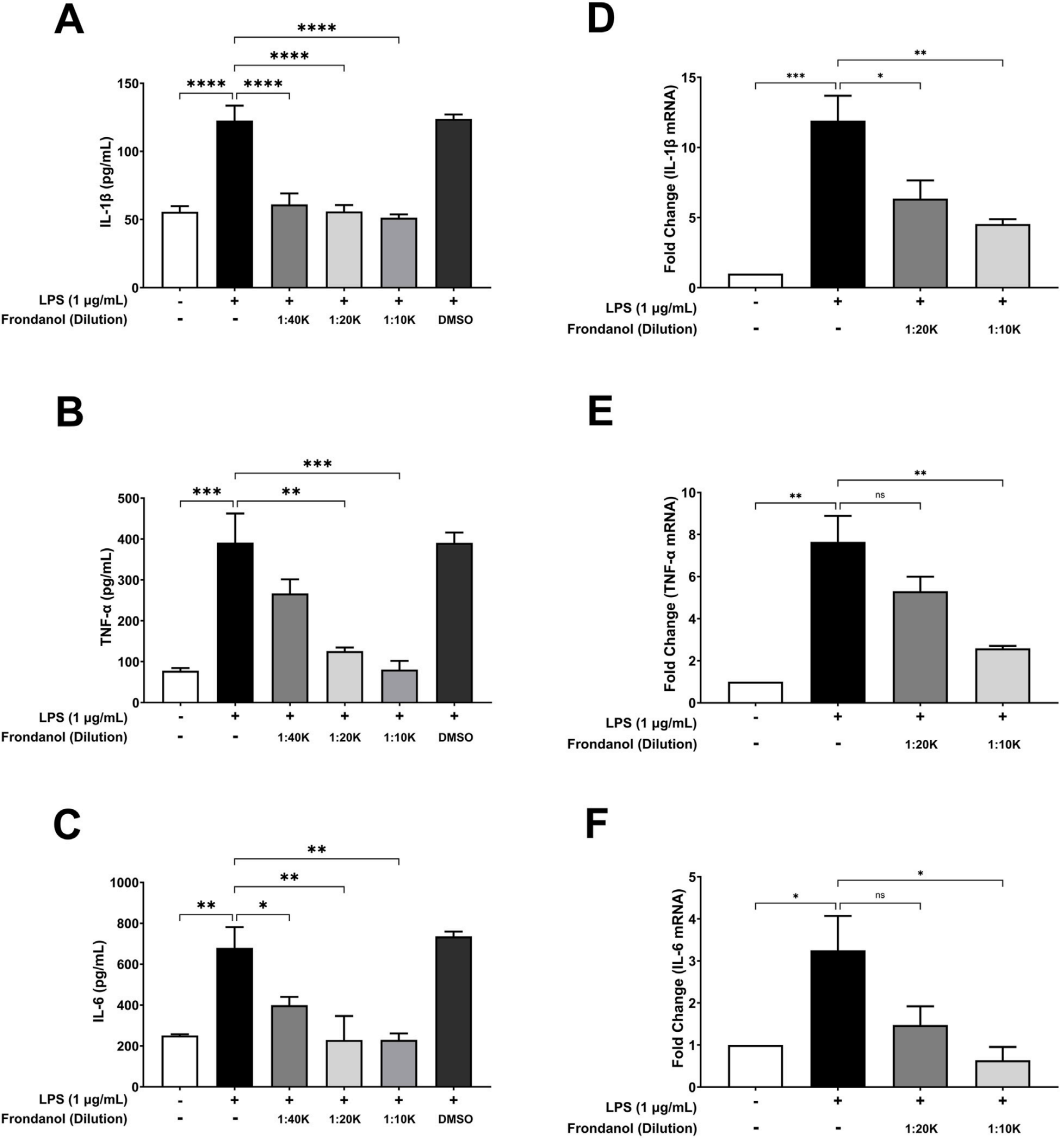
Effects of Frondanol on LPS-induced proinflammatory cytokine **(A–C)** productions and their respective mRNA expression **(D–F)** in RAW 264.7 macrophages. Data are expressed as mean ± SEM of three independent experiments. *p < 0.05, **p < 0.01, ***p < 0.001, and ***p < 0.0001.

To evaluate the anti-inflammatory effects of Frondanol, we analyzed its impact on LPS-induced pro-inflammatory cytokine secretion in RAW 264.7 macrophages. ELISA quantification showed that LPS stimulation significantly increased the secretion of IL-1β, TNF-α, and IL-6 (Figures 4A–C). Treatment with Frondanol at different dilutions (1:40 K, 1:20 K, 1:10 K) resulted in a marked reduction in these cytokines compared to the LPS model group (p < 0.01 to p < 0.0001). The highest dilution (1:10 K) demonstrated the most significant inhibitory effect, comparable to the untreated control. Since the 1:40 K dilution did not show a marked reduction in TNF-α level, it was excluded from subsequent qPCR analysis. Furthermore, gene expression analysis using qPCR confirmed that Frondanol suppressed LPS-induced expression of key inflammatory markers. The relative mRNA levels of IL-1β, TNF-α, and IL-6 were significantly upregulated in response to LPS stimulation (Figures 4D–F). Treatment with Frondanol at 1:20 K and 1:10 K dilutions significantly downregulated these inflammatory genes, with the highest inhibition observed at the 1:10 K dilution. These findings suggest that Frondanol exerts potent anti-inflammatory effects by reducing LPS-induced cytokine production and suppressing pro-inflammatory gene expression.

### 3.6 Frondanol attenuates the activation and translocation of NF-κB

**FIGURE 5:**
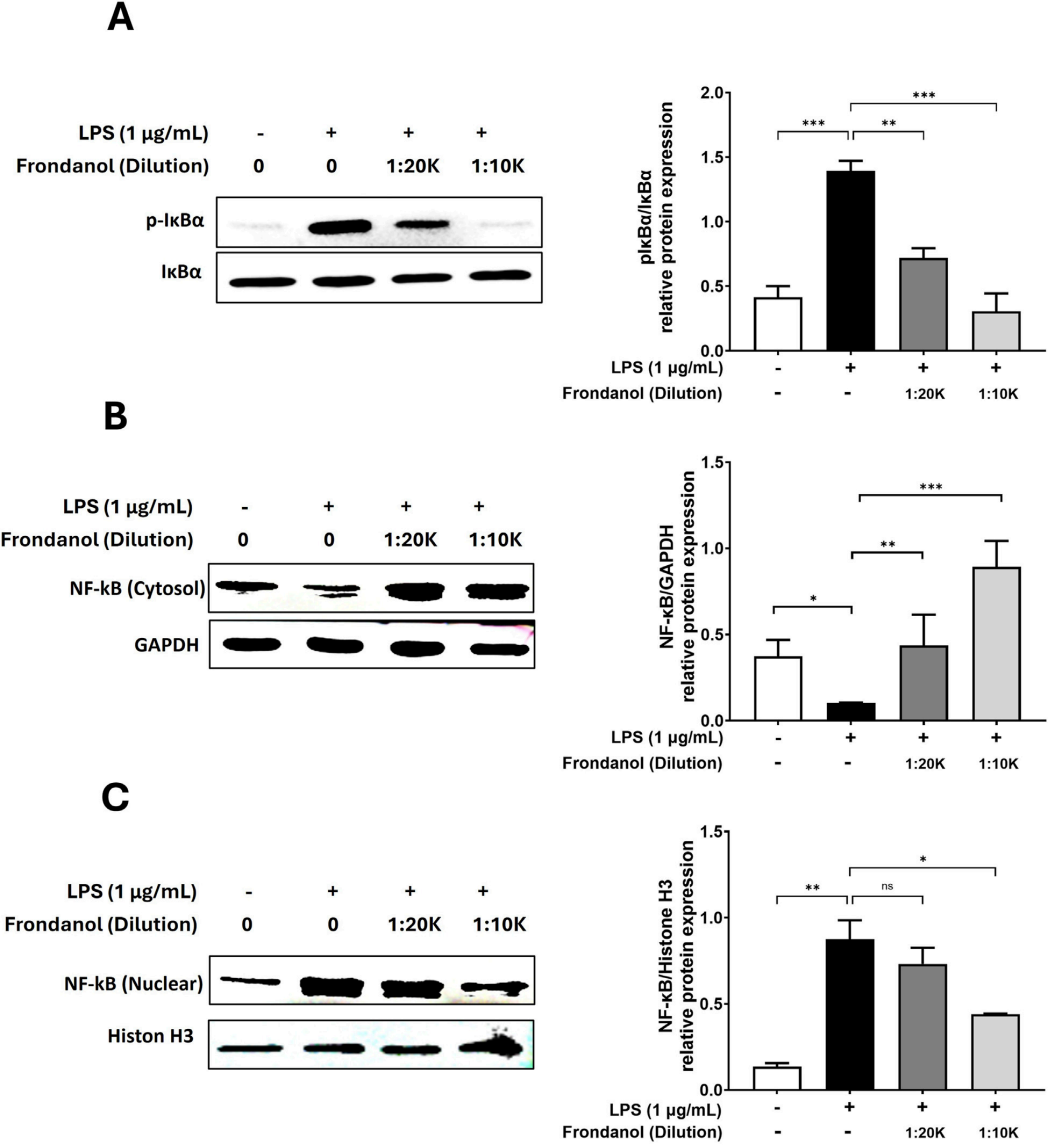
The inhibitory effects of Frondanol on NF-κB activation and nuclear translocation. Frondanol inhibited LPS-induced NF-κB activation by reducing IκBα phosphorylation and preventing p65 nuclear translocation. **(A)** Phosphorylation of IκBα was assessed by Western blot analysis in total cell lysates, with p-IκBα expression normalized to total IκBα. **(B,C)** NF-κB p65 translocation was analyzed by detecting its levels in nuclear and cytoplasmic fractions. Representative Western blot images from two to three independent experiments are shown. The intensity of protein bands was quantified and expressed as bar graphs. Data are presented as the means ± SEM of three independent experiments. *p < 0.05, **p < 0.01, ***p < 0.001, and ****p < 0.0001.

To assess the effect of Frondanol on LPS-induced NF-κB activation, we examined the translocation of NF-κB p65 subunit from the cytoplasm to the nucleus. Additionally, we analyzed the phosphorylation of IκBα to understand the upstream signaling events. As shown in Figure 5A, LPS stimulation significantly increased the phosphorylation of IκBα compared to the untreated control, leading to NF-κB activation. However, Frondanol treatment at both 1:20 K and 1:10 K dilutions reduced p-IκBα levels, suggesting inhibition of NF-κB activation. Figure 5C demonstrates that LPS stimulation markedly increased nuclear p65 levels, indicating its translocation from the cytoplasm (Figure 5B) to the nucleus. In contrast, Frondanol treatment effectively reduced nuclear p65 levels while increasing cytoplasmic p65, suggesting inhibition of NF-κB nuclear translocation.

### 3.7 Frondanol downregulates MAPK pathway protein expressions

**FIGURE 6:**
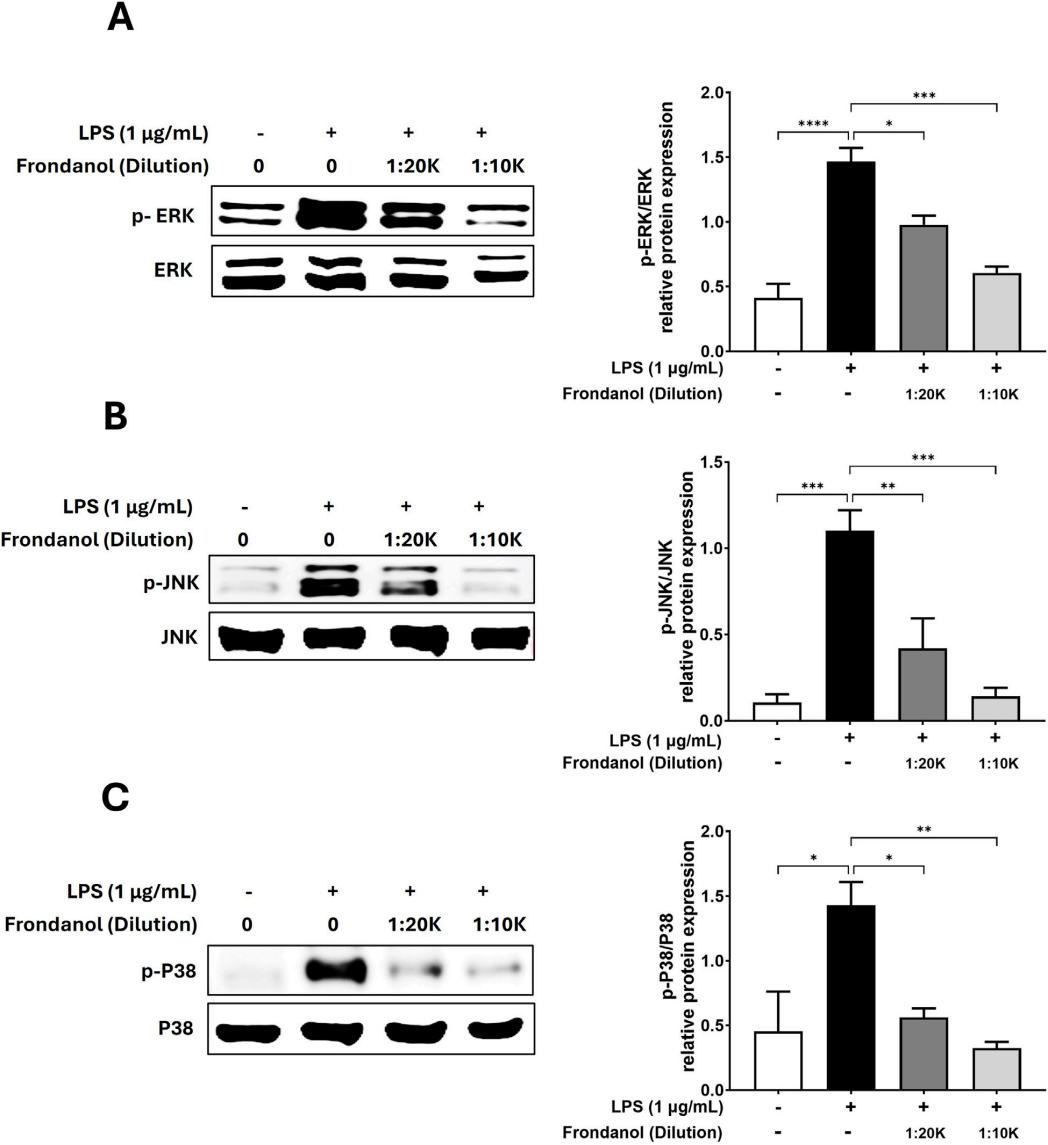
The inhibitory effects of Frondanol on the activation of MAPK. Frondanol inhibited the phosphorylation of MAPK, including ERK, p38, and JNK. Total cell lysates were used, and the expression levels of **(A)** ERK, phospho-ERK, **(B)** JNK, phospho-JNK, **(C)** p38, and phospho-p38 were determined by Western blot analysis. p-MAPK expression was normalized to the corresponding total MAPK level. The images shown are representative of three independent experiments with similar results. The intensity of protein bands from the western blots was quantified and expressed as bar graphs. Data are expressed as the means ± SEM of three independent experiments. *p < 0.05, **p < 0.01, ***p < 0.001 and ****p < 0.0001.

To evaluate the anti-inflammatory effects of Frondanol, we examined its impact on the phosphorylation of ERK, JNK, and p38 MAPK in LPS-stimulated cells. Western blot analysis demonstrated that LPS (1 μg/mL) treatment significantly increased ERK, JNK, and p38 phosphorylation levels compared to the untreated control. However, pretreatment with Frondanol at 1:20 K and 1:10 K dilutions reduced the phosphorylation of these MAPK pathway components in a dose-dependent manner (Figure 6).

## 4 Discussion

Several studies have explored the health benefits of naturally derived compounds and extract due to their widespread availability and minimal toxicity. While numerous compounds and extracts derived from traditional medicinal plants and marine organisms have demonstrated anti-inflammatory properties, few studies have investigated the molecular mechanisms underlying the anti-inflammatory effects of those extracts and compounds. In this study, we investigated the anti-inflammatory effects of Frondanol, a nutraceutical extract derived from the sea cucumber Cucumaria frondosa, on LPS-induced RAW 264.7 macrophages. Our findings demonstrated that Frondanol effectively attenuated LPS-induced inflammatory responses by modulating key signalling pathways, including NF-κB and MAPK, and by suppressing the production of pro-inflammatory mediators such as NO, PGE2, and cytokines (IL-1β, TNF-α, and IL-6). These findings suggest that Frondanol may hold potential as a natural anti-inflammatory compound, warranting further investigation in animal and clinical models.

Activated macrophages produce excessive inflammatory mediators, such as NO and PGE2, along with proinflammatory cytokines like IL-1β and IL-6, TNF-α, which drive the inflammatory response and interact synergistically with other mediators (Medzhitov, 2008; Chen et al., 2023). Marine-derived compounds and extracts capable of reducing these inflammatory factors are of significant interest as potential anti-inflammatory agents. Consequently, extensive research has focused on evaluating the inhibitory effects of marine compounds on NO and PGE2 to develop therapeutic strategies for inflammatory diseases (Vasarri and Degl'Innocenti, 2024; Qiu et al., 2024; Khursheed et al., 2023). Several studies indicate that natural compounds derived from marine organisms effectively reduce inflammation by inhibiting the transcription of iNOS and COX-2 (Ghelani et al., 2022; Khursheed et al., 2023). In our study, LPS stimulation significantly increased NO and PGE2 production, along with the upregulation of iNOS and COX-2 expression at both the mRNA and protein levels. However, treatment with Frondanol markedly reduced these effects in a dose-dependent manner. The reduction in NO and PGE2 levels, along with the downregulation of iNOS and COX-2 expression, suggests that Frondanol exerts its anti-inflammatory effects by inhibiting the synthesis of these key inflammatory mediators.

Furthermore, pro-inflammatory cytokines such as IL-1β, IL-6 and TNF-α play a central role in the amplification of inflammatory responses and are implicated in the development of various inflammatory diseases (Kany et al., 2019; Neurath, 2014). Our results show that LPS stimulation significantly increased the secretion of these cytokines, which was effectively suppressed by Frondanol treatment. Furthermore, Frondanol downregulated the mRNA expression of these cytokines, indicating that it acts at the transcriptional level to inhibit their production.

NF-κB is a transcription factor that regulates the expression of inflammatory mediator genes including iNOS, COX-2 and pro-inflammatory cytokines. Under normal conditions, it remains inactive in the cytoplasm, bound to its inhibitory subunit, IκBα. Upon stimulation with LPS, IκBα undergoes phosphorylation, triggering its proteolytic degradation. This process facilitates the translocation of NF-κB into the nucleus, where it activates gene transcription (Medzhitov, 2008; Tak and Firestein, 2001). In our study, we observed that the upregulation of phosphorylated IκBα by LPS stimulation was effectively reduced by Frondanol treatment. Moreover, Western blot experiments revealed that the nuclear translocation of NF-κB was significantly reduced by Frondanol, further confirming Frondanol’s NF-κB inhibitory activity.

Similar to other natural products with known anti-inflammatory properties—such as anthocyanins (Garcia et al., 2021), carvacrol (Somensi et al., 2019), and the flavanol-rich butanol fraction of *Uvaria alba* (Notarte et al., 2023)*,* Frondanol also exerts its effects through the inhibition of the NF-κB signaling pathway. Moreover, while Notarte et al. (2023). demonstrated that the flavonol-rich butanol fraction of *Uvaria alba* modulates both the NF-κB and NRF2 pathways, our study specifically focuses on the modulation of NF-κB and MAPK signaling pathways. Although NRF2 activity was not assessed in the current work, it remains a valuable target for future investigation—particularly considering the key role of oxidative stress in the pathogenesis of chronic inflammatory conditions.

The activation of NF-κB is regulated by multiple cellular kinases, including MAPKs which play crucial role in inflammatory responses. MAPKs are key components of pro-inflammatory signaling pathways, and studies have shown that the activation of JNK, p38 MAPK, and ERK contributes to the upregulation of iNOS and COX-2 by promoting NF-κB activation in LPS-stimulated immune cells (Guo et al., 2024). Consequently, the suppression of MAPK phosphorylation in activated RAW 264.7 cells is closely associated with anti-inflammatory mechanisms (Guo et al., 2024; Kwon et al., 2013; Yoon and Park, 2021). We observed that Frondanol attenuated the phosphorylation of ERK1/2, JNK, and p38 MAPK, which are key components of the MAPK signaling pathway in LPS stimulated RAW 264.7 macrophage.

The fatty acid profile of Frondanol demonstrates a strong potential for anti-inflammatory activity. 12-MTA, the most abundant branch-chain fatty acid in Frondanol, is known for its role in immune regulation and inflammation. *In vivo* and *in vitro* studies have shown that 12-MTA selectively inhibits the 5-lipoxigenase pathway, reducing the pro-inflammatory eicosanoids, 5-HETE and leukotriene B4 (Yang et al., 2003). Furthermore, *in vivo* arterial delivery of 12-MTA in rabbit tumor model also showed a reduction in levels of 5-HETE, with a concomitant increase in levels of the anti-inflammatory eicosanoid 15-HETE suggesting modulation of both of these lipoxygenase pathways (Wright et al., 2005). More recent reviews describe 12-MTA as a part of dietary branch chain fatty acids which downregulate pro-inflammatory gene expression in adipocytes and hepatocytes (Czumaj et al., 2022). These studies support 12-MTA’s role as a bioactive fatty acid which can attenuate the inflammation by modulating eicosanoid pathways and inflammatory gene regulation. Notably, Frondanol also contains high levels of polyunsaturated fatty acids particularly EPA. EPA is well-documented for its potent anti-inflammatory and immunomodulatory effects. EPA competitively inhibits the synthesis of pro-inflammatory arachidonic acid-derived eicosanoids, further impairing the production of pro-inflammatory prostaglandins and leukotrienes (Fernández-Lázaro et al., 2024). At the cellular level, EPA integrates into the phospholipid membrane and inhibits NF-κB (reducing inflammatory gene expression) and activating PPAR-γ expression (enhancing anti-inflammatory gene expression) (Calder, 2017). Several clinical and animal studies also demonstrated the therapeutic benefits of EPA in various inflammatory conditions such as rheumatoid arthritis and atherosclerosis (Calder, 2017). Thus, fatty acid profiling data suggests that 12-MTA, and EPA be major bioactive lipids responsible for the observed anti-inflammatory activity of Frondanol.

The anti-inflammatory effects of Frondanol observed in this study are consistent with previous reports demonstrating its efficacy in animal models of colitis and other inflammatory conditions (Subramanya et al., 2018; Collin, 2002). The ability of Frondanol to modulate multiple inflammatory pathways, including NF-κB and MAPK, and to suppress the production of key inflammatory mediators and cytokines, highlights its potential as a therapeutic agent for chronic inflammatory diseases. Moreover, the lack of cytotoxicity at the tested concentrations suggests that Frondanol is safe for further development as a nutraceutical or adjunct therapy.

While our study provides valuable insights into the anti-inflammatory mechanisms of Frondanol, there are some limitations. First, the study was conducted *in vitro* using a murine macrophage cell line, which may not fully replicate the complexity of human inflammatory responses. Future studies should investigate the effects of Frondanol *in vivo* using animal models of chronic inflammatory diseases such as rheumatoid arthritis, to validate its therapeutic potential. To rule out the possibility that the solvent (DMSO) contributed to the observed anti-inflammatory effects, we included 0.5% DMSO as a vehicle control in all experiments. This concentration corresponds to the DMSO content present in the highest dilution of Frondanol tested (1:10 K). Our results showed that DMSO alone did not produce any significant reduction in inflammatory markers such as NO, PGE_2_, iNOS, COX-2, or pro-inflammatory cytokines. Therefore, it is reasonable to conclude that DMSO had no or minimal contribution to the anti-inflammatory activity observed with Frondanol treatment in this study.

**FIGURE 7:**
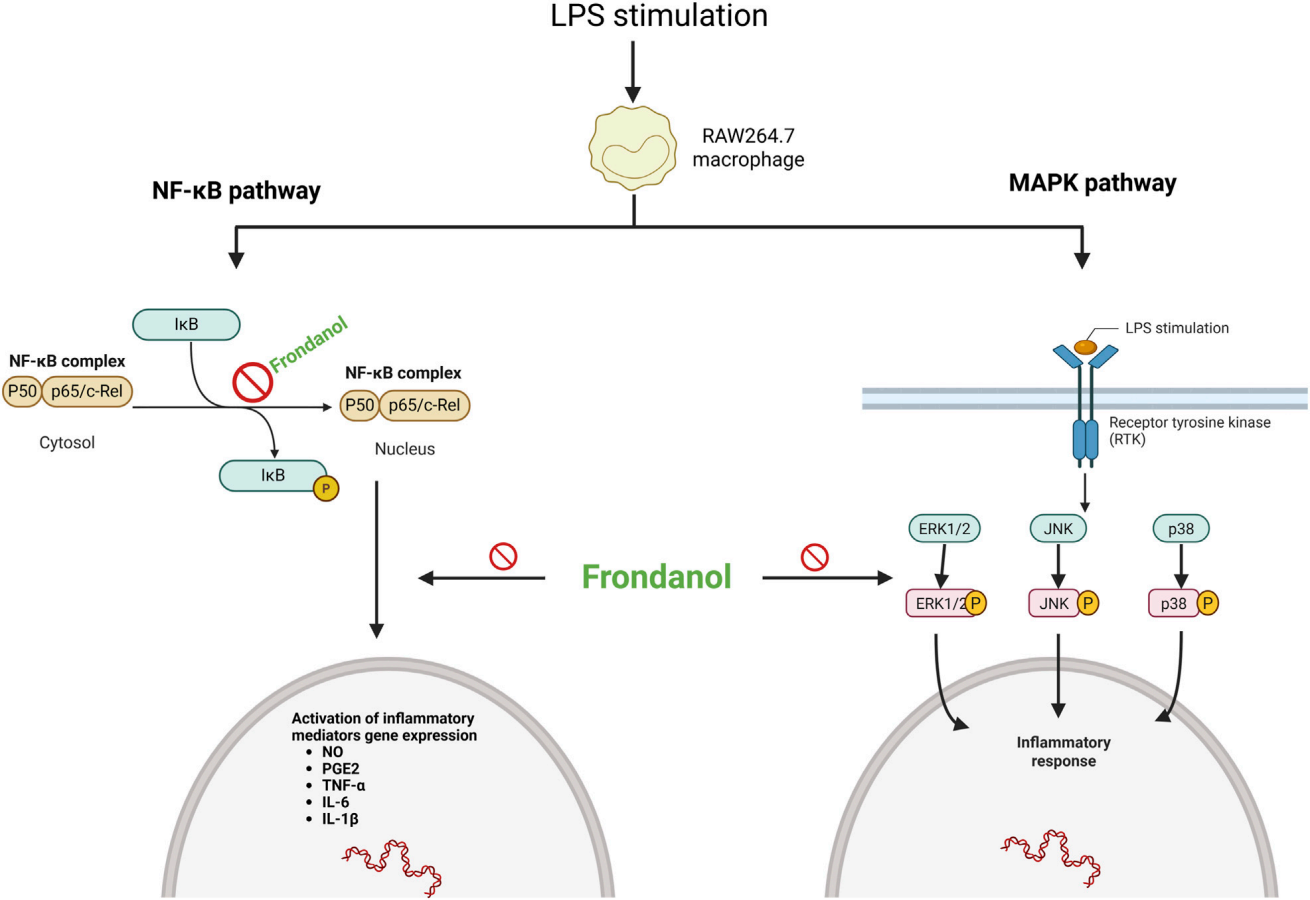
Schematic representation of Frondanol’s proposed anti-inflammatory mechanism via inhibition of NF-κB and MAPK pathways in LPS-stimulated RAW 264.7 macrophages. (created on Bio Render).

In conclusion, our study demonstrates that Frondanol exerts potent anti-inflammatory effects in LPS-induced RAW 264.7 macrophages by modulating the NF-κB and MAPK signalling pathways and suppressing the production of inflammatory mediators and cytokines (Figure 7). These findings suggest that Frondanol may have potential as a therapeutic candidate for the prevention and treatment of chronic inflammatory diseases, although further studies are warranted to confirm its efficacy *in vivo* and to identify the specific bioactive compounds responsible for its anti-inflammatory properties.

## Data Availability

The original contributions presented in the study are included in the article/supplementary material, further inquiries can be directed to the corresponding author.
